# Flexibility in the ambrosia symbiosis of *Xyleborus bispinatus*

**DOI:** 10.3389/fmicb.2023.1110474

**Published:** 2023-03-02

**Authors:** Octavio Menocal, Luisa F. Cruz, Paul E. Kendra, Marielle Berto, Daniel Carrillo

**Affiliations:** ^1^Tropical Research and Education Center, University of Florida, Homestead, FL, United States; ^2^United States Department of Agriculture, Agricultural Research Service, Subtropical Horticulture Research Station, Miami, FL, United States

**Keywords:** ambrosia beetles, flexible symbiosis, fungal partners, *Fusarium*, *Harringtonia lauricola*, laurel wilt, mutualism, *Raffaelea lauricola*

## Abstract

**Introduction:**

Ambrosia beetles maintain strict associations with specific lineages of fungi. However, anthropogenic introductions of ambrosia beetles into new ecosystems can result in the lateral transfer of their symbionts to other ambrosia beetles. The ability of a Florida endemic ambrosia beetle, *Xyleborus bispinatus*, to feed and establish persistent associations with two of its known symbionts (*Raffaelea subfusca* and *Raffaelea arxii*) and two other fungi (*Harringtonia lauricola* and *Fusarium* sp. nov.), which are primary symbionts of invasive ambrosia beetles, was investigated.

**Methods:**

The stability of these mutualisms and their effect on the beetle’s fitness were monitored over five consecutive generations. Surface-disinfested pupae with non-developed mycangia were reared separately on one of the four fungal symbionts. Non-treated beetles (i.e., lab colony) with previously colonized mycangia were used as a control group.

**Results:**

*Xyleborus bispinatus* could exchange its fungal symbionts, survive, and reproduce on different fungal diets, including known fungal associates and phylogenetically distant fungi, which are plant pathogens and primary symbionts of other invasive ambrosia beetles. These changes in fungal diets resulted in persistent mutualisms, and some symbionts even increased the beetle’s reproduction. Females that developed on *Fusarium* sp. nov. had a significantly greater number of female offspring than non-treated beetles. Females that fed solely on *Harringtonia* or *Raffaelea* symbionts produced fewer female offspring.

**Discussion:**

Even though some ambrosia beetles like *X. bispinatus* can partner with different ambrosia fungi, their symbiosis under natural conditions is modulated by their mycangium and possibly other environmental factors. However, exposure to symbionts of invasive beetles can result in stable partnerships with these fungi and affect the population dynamics of ambrosia beetles and their symbionts.

## 1. Introduction

Ambrosia beetles (Coleoptera: Curculionidae: Scolytinae and Platypodinae) live in obligate nutritional relationships with ambrosia fungi (Farrell et al., 2001; Mueller et al., 2005; Biedermann and Vega, 2020). In this mutualistic relationship, the fungi serve as food sources for the beetles, whereas the fungi rely on the beetles for protection, dispersal, and maintenance (Biedermann and Taborsky, 2011; Hulcr and Stelinski, 2017; Nuotclà et al., 2019). Some ambrosia beetles are associated with a single dominant mutualistic fungus, while others are associated with multiple fungal symbionts (Batra, 1963; Gebhardt et al., 2004; Harrington et al., 2010; Kostovcik et al., 2015; Menocal et al., 2017, 2018a; Cruz et al., 2018, 2019, 2021; Saucedo-Carabez et al., 2018; Carrillo et al., 2019). Females in the *Xylosandrus* and *Euwallacea* species-complex are commonly associated with *Ambrosiella* (Microascales: Ceratocystidaceae) and *Fusarium* (Hypocreales: Nectriaceae) species, respectively, whereas females in the genera *Xyleborinus* and *Xyleborus* are strictly associated with *Raffaelea* species (Ophiostomatales: Ophiostomateacea), suggesting that ambrosia beetles exhibit fidelity toward specific groups of fungal symbionts (Biedermann et al., 2009, 2013; Kasson et al., 2013; Bateman et al., 2015; Mayers et al., 2015, 2020a,b; O’Donnell et al., 2015; Biedermann, 2020).

A few studies have investigated ambrosia beetle symbiont exchange or transition to specific fungi. Carrillo et al. (2014) reported for the first time the lateral transfer of nutritional symbionts between native and exotic ambrosia beetles. Skelton et al. (2019) documented that *Xylosandrus compactus* Eichhoff can exchange nutritional fungal symbionts among closely related *Ambrosiella* species, but a shift to more phylogenetically distant fungal species is less likely to occur. In a different study, Carrillo et al. (2020a) demonstrated that various shot hole borers (*Euwallacea* spp.) can survive and reproduce using each other’s *Fusarium* fungal symbionts. These studies suggest that transition to other nutritional fungal symbionts could occur more commonly among congeneric ambrosia beetle species that share closely related fungi and in species with relatively small mycangia [pre-oral (*Xyleborus* spp.) vs. mesonotal mycangia (*Xylosandrus* spp.)] (Joseph and Keyhani, 2021; Mayers et al., 2022).

Acquisition of new fungal symbionts may occur naturally when one ambrosia beetle species interacts with the fungi inside the gallery system of another species or, when mature females that search for new hosts, encounter fungi present in the environment (Kendra et al., 2011; Carrillo et al., 2014; Rassati et al., 2019). However, only the first mechanism is expected to involve nutritional fungal symbionts. Nutritional ambrosia fungi are not directly transmitted from mother to offspring. Larvae grow feeding on symbiotic fungi, but their gut contents are emptied during the pupal stage. Then, naïve adults acquire their symbionts *via* feeding (*Xyleborus* and *Euwallacea*) or by close contact with the gallery walls (*Xylosandrus*). This period of independent growth is a critical point for the acquisition of new nutritional fungal symbiont (s) (Six, 2012).

Some invasive ambrosia beetles partner with plant pathogens that can seriously damage forest and agricultural systems (Hanula et al., 2008; Hulcr and Dunn, 2011; Six, 2012; Ploetz et al., 2013; Hughes et al., 2017; Hulcr and Stelinski, 2017). Two new diseases currently threaten avocado production in South Florida. Laurel wilt (LW) and *Fusarium* dieback (FD) are caused by *Harringtonia lauricola* (T. C. Harr, Fraedrich & Aghayeva) Z. W. de Beer & M. Procter and *Fusarium* sp. nov., primary symbionts of *Xyleborus glabratus* Eichhoff and *Euwallacea perbrevis* Schedl, respectively (Harrington and Fraedrich, 2010; Eskalen et al., 2013; Smith et al., 2019; de Beer et al., 2022). Although *X. glabratus* is rarely detected in avocado orchards, other native and exotic ambrosia beetles in these orchards have acquired *H. lauricola via* lateral transfer (Carrillo et al., 2014; Kendra et al., 2020; Cloonan et al., 2022). The LW pathogen is now associated with the mycangial communities of several *Xyleborus*, *Ambrosiodmus*, *Xyleborinus*, and *Xylosandrus* species (Carrillo et al., 2014; Ploetz et al., 2017; Cruz et al., 2021). Unlike *H. lauricola* and its secondary vectors, the FD pathogen (s) are only vectored by *Euwallacea* spp. However, experiments evaluating the interaction between *Fusarium* spp. and other Scolytinae species outside the genus *Euwallacea* are lacking.

Florida avocado orchards house a diverse community of ambrosia beetles that often breed in trees affected by either LW or FD (Carrillo et al., 2012, 2016; Kendra et al., 2017, 2020; Menocal et al., 2018b, 2022; Owens et al., 2019a,b; Rivera et al., 2020; Cloonan et al., 2022). One of these species, *Xyleborus bispinatus* Eichhoff, is a Florida native beetle that has shown plasticity in its fungal symbiosis (Cruz et al., 2018). This species has become relevant due to its frequent partnership with *H. lauricola*, and its ability to transmit the pathogen to healthy avocado trees (Carrillo et al., 2014). However, the persistence of this association, the possible additional association with FD pathogen (s), and the effect of these pathogens on the beetle’s fitness are still understudied. To gain insights into this novel partnership, the symbionts of *X. bispinatus* were removed and the symbiosis was re-established with selected ambrosia fungi species. The specific objectives of this study were to: (1) determine whether *X. bispinatus* can feed and reproduce on *H. lauricola, Fusarium* sp. nov., and its original nutritional symbionts *R. subfusca* and *R. arxii*, (2) determine how stable these associations could be over several generations, and (3) determine the effect that each fungal symbiont has on offspring production.

## 2. Materials and methods

### 2.1. Beetle collection, laboratory rearing, and aposymbiotic beetles

*Xyleborus bispinatus* adult females were collected *in-flight* at dusk from an avocado orchard (N25° 35′42” W80° 28′ 21″) (Kendra et al., 2012). Briefly, a white cotton fabric (3 × 3 m) was placed on the ground, and ethanol lures (ultra-high release; Contech Enterprises, Inc., Victoria, BC, Canada) were hung from a tripod at ~1 m above the fabric. Beetles landing on the fabric were collected and placed into plastic containers with moist tissue paper for transport to the laboratory. Then, all females were surface disinfested in 70% ethanol for 10 s to reduce contaminants that could jeopardize laboratory rearing. More active and vigorous females were introduced individually into rearing tubes filled with sterile avocado sawdust artificial media to start new colonies (Menocal et al., 2017, 2018a). Rearing tubes were stored in a walk-in rearing room (25 ± 1°C, 70% RH) for 24 days, allowing the new progeny to reach the pupal stage (Cruz et al., 2018). Then, colonies were dissected under sterile conditions in a laminar flow hood by systematically cutting the rearing media plug into small pieces. Gallery tunnels were opened carefully by removing the medium around them. All male and female pupae from a single colony were gently removed and placed into a sterilized Petri dish. Pupae were individually transferred to Eppendorf tubes to be surface-disinfested by a sequence of washes with phosphate-buffered saline (PBS) + 0.1% Tween, 2% sodium hypochlorite, 70% ethanol, and sterile water for 10, 5, 10, and 10 s, respectively. After the washes, presumably aposymbiotic pupae from each colony were regrouped and allowed to dry before being exposed to selected fungi.

### 2.2. Fungal isolates, growth conditions, and development of *Xyleborus bispinatus* on single fungal symbionts

*Harringtonia lauricola*, *R. subfusca*, and *R. arxii* isolates recovered from *X. bispinatus* and a *Fusarium* sp. isolate recovered from *E. perbrevis* were used in the experiment (Table 1). Spore solutions of each fungus were obtained by gently scraping two-week-old monosporic cultures with a sterile plastic rod (Fisher Scientific Catalog No. 23600896) and resuspending them in sterile water to a final concentration of 1 × 10^6^ conidia/mL. The spore solutions (100 μl) were individually plated on 47-mm Petri dishes containing the same avocado sawdust media used to rear the beetles. These cultures were incubated at 25°C for 10 days in complete darkness until the avocado sawdust media was colonized entirely. Then, groups of one male and ten female aposymbiotic pupae from the same colony were transferred to the Petri dishes with the fungal cultures and maintained in complete darkness at room temperature (25°C). Several holes, 0.5 cm deep, were made on the surface of the artificial substrate to promote boring by emerging adults. Adults remained in the plates for 7–10 days to complete sclerotization, fungal acquisition, and mating. Afterward, 20 active, vigorous, and presumably fertilized females, exposed to a given fungal species were placed individually in rearing tubes with sterile avocado sawdust media to establish new colonies. Non-altered *X. bispinatus* colonies (i.e., with non-modified symbionts) containing *R. arxii*, *R. subfusca*, *R. subalba*, and numerous yeasts, but not *H. lauricola* (Cruz et al., 2018) were used as the control treatment. All colonies were dissected 40 days later to extract 20 new fully sclerotized females to start new colonies. This procedure was repeated until completing five generations. The number of female and male offspring was recorded for each colony of each generation.

**Table 1:** GenBank accession numbers and primers used for identification of four fungal symbionts used as diets by *Xyleborus bispinatus* in this study.

Symbiont	Locus	GenBank accession no.	Primers	References
*Harringtonia lauricola*	28S rDNA LSU	MT556279	LR0R/LR5	Vilgalys and Hester (1990)
	18S rDNA SSU	HM446155	NS1/NS4	White et al. (1990)
*Raffaelea subfusca*	28S rDNA LSU	OK324060	LR0R/LR5	Vilgalys and Hester (1990)
	18S rDNA SSU	OK324047	NS1/NS4	White et al. (1990)
*Raffaelea arxii*	28S rDNA LSU	OK324059	LR0R/LR5	Vilgalys and Hester (1990)
	18S rDNA SSU	OK324046	NS1/NS4	White et al. (1990)
*Fusarium* sp. nov.	EF1-α	MZ265349	EF1	O’Donnell et al. (1998)
	RPB1	MZ265352	F5/R8; F8/R9	O’Donnell et al. (2010)
	RPB2	MZ265355	5f2/7cr; 7cf/11ar	Liu and Hall (2004) and Reeb et al. (2004)

### 2.3. Fungal symbiont recovery, quantification, DNA extraction, and PCR amplification

One female offspring from each colony and each generation was assayed for fungal presence. Briefly, females were surface disinfested in 70% ethanol, agitated for 10 s, and subsequently rinsed in sterile deionized water. Then, females were individually macerated in 500 μl of sterile water. Aliquots of 100 μl of the macerate were plated on cycloheximide-streptomycin-malt agar (CSMA), a selective medium for Ophiostomatales (Harrington et al., 2010), and potato dextrose agar amended with 0.1 g/l streptomycin (PDA^+^). Plates were incubated for 5–7 days at 25°C in complete darkness. The number of colony-forming units (CFUs) was recorded for each colony in each generation. Plates exhibiting contaminants such as *Aspergillus* spp., *Penicillium* spp., and/or more than one fungal phenotype were discarded along with its corresponding rearing tube. Plates displaying phenotypically identical CFUs were subcultured to obtain four to six single-spore cultures. DNA was extracted from the mycelium of monosporic isolates following a modified Cetyltrimethylammonium Bromide (CTAB) protocol (Doyle and Doyle, 1987). Fungal symbionts were identified using the nuclear ribosomal large subunit (28S), the nuclear ribosomal small subunit (18S), translation elongation factor 1-alpha (EF1α), RNA polymerase largest subunit (RPB1), and RNA polymerase second largest subunit (RPB2). Primers are listed in Table 1. In addition, the identity of *H. lauricola* was confirmed with two diagnostic microsatellite markers, CHK and IFW (Dreaden et al., 2014). PCR products were purified using ExoSAP-IT (Affymetrix, CA, USA) following the manufacturer’s guidelines. Sequencing of the PCR products was carried out by Eurofins genomics (Louisville, KY).

### 2.4. Statistical analysis

Data were analyzed using SAS v. 9.4 (PROC GLIMMIX, SAS Institute 2010, Cary, NC, USA). Separate one-way analyses of variance (ANOVA) per fungal symbiont were conducted to detect differences in the number of CFUs and female offspring among generations. Number of males were too few for statistical analysis. A negative binomial distribution was used when analyzing CFU data due to overdispersion in the dataset, and a Poisson distribution was used for female offspring production. Due to the non-normality of the data set and variance heterogeneity, separate Kruskal-Wallis tests were used to detect differences in female offspring production between each fungal symbiont against non-treated beetles in each generation. To determine which fungal symbiont supported more *X. bispinatus* progeny, female offspring were compared among fungal treatments through an analysis of variance (ANOVA) with a Poisson distribution. Finally, any significant differences were identified by multiple post-hoc comparisons (Tukey’s HSD test) at *p* < 0.05. All figures were generated using JMP Pro 14.

## 3. Results

**Figure 1:**
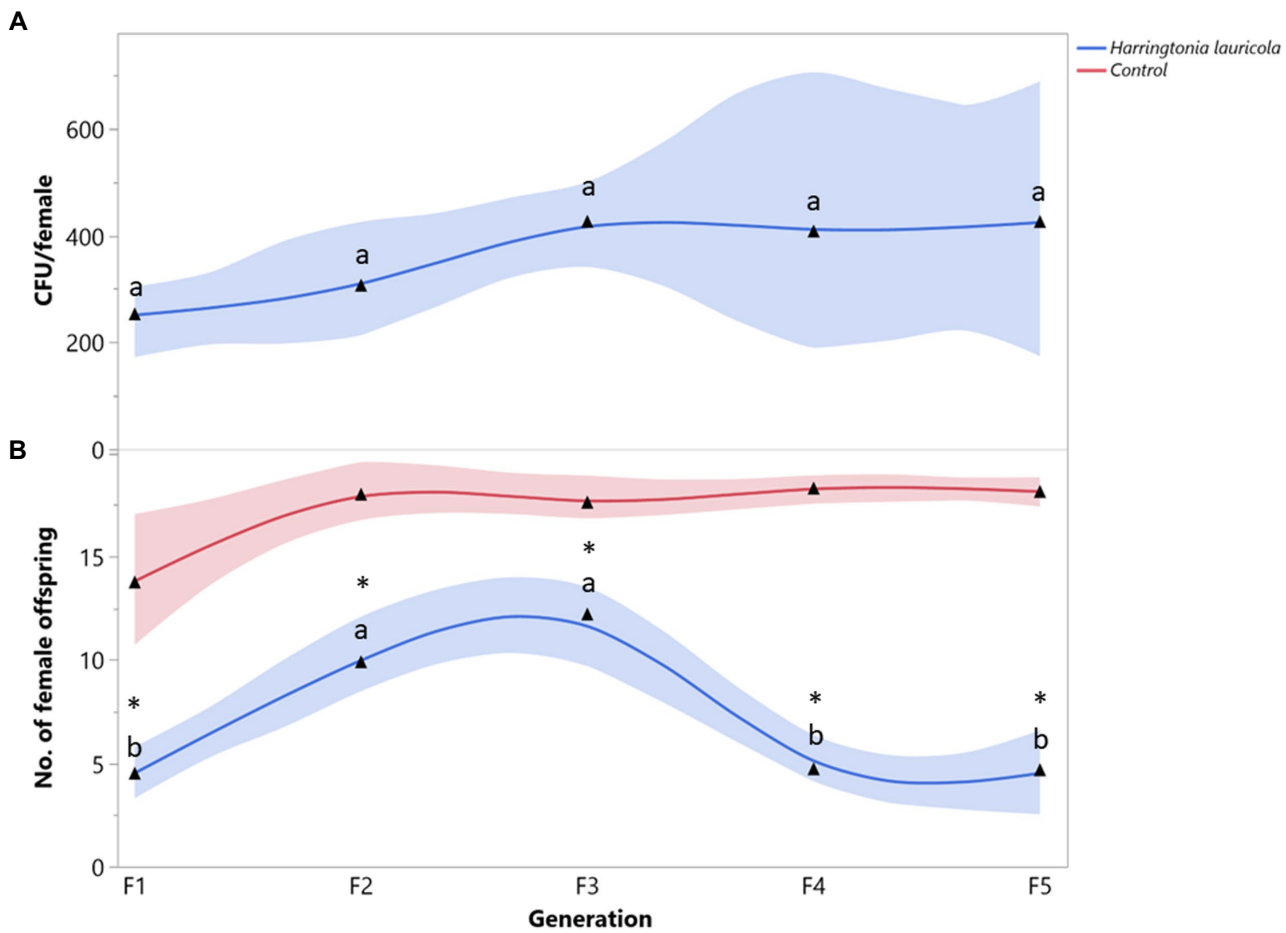
*Xyleborus bispinatus* performance with a single mutualistic fungus (*Harringtonia lauricola*). **(A)** Mean number of CFUs (▲) per female offspring per generation and **(B)** Mean number of female offspring (▲) produced by single female founder per generation. Same letters within the same color are not significantly different (Tukey HSD, α = 0.05). Asterisks show significant differences in female offspring between fungal symbiont and control within generation (Kruskall-Wallis, α = 0.05).

The mean number of *H. lauricola* propagules recovered from *X. bispinatus* was relatively constant among generations (*F* = 0.4752; df = 4, 28; *p* = 0.7218) (Figure 1A). Females fed solely on *H. lauricola* increased reproduction (i.e., female offspring) in the first three generations and declined drastically in the last two (*F* = 9.4419; df = 4, 28; *p* < 0.0001) (Figure 1B). Switching to a *H. lauricola* diet resulted in significantly fewer female offspring than that recorded from control females with unaltered symbiosis throughout the five generations (F_1_: *X^2^* = 9.3272, *p* = 0.0023; F_2_: *X^2^* = 11.2295, *p* = 0.0008; F_3_: *X^2^* = 7.4715, *p* = 0.0063; F_4_: *X^2^* = 14.4687, *p* = 0.0023; F_5_: *X^2^* = 12.4670, *p* = 0.0004) (Figure 1B). Notably, no individuals fed exclusively on *H. lauricola* survived after the fifth generation.

**Figure 2:**
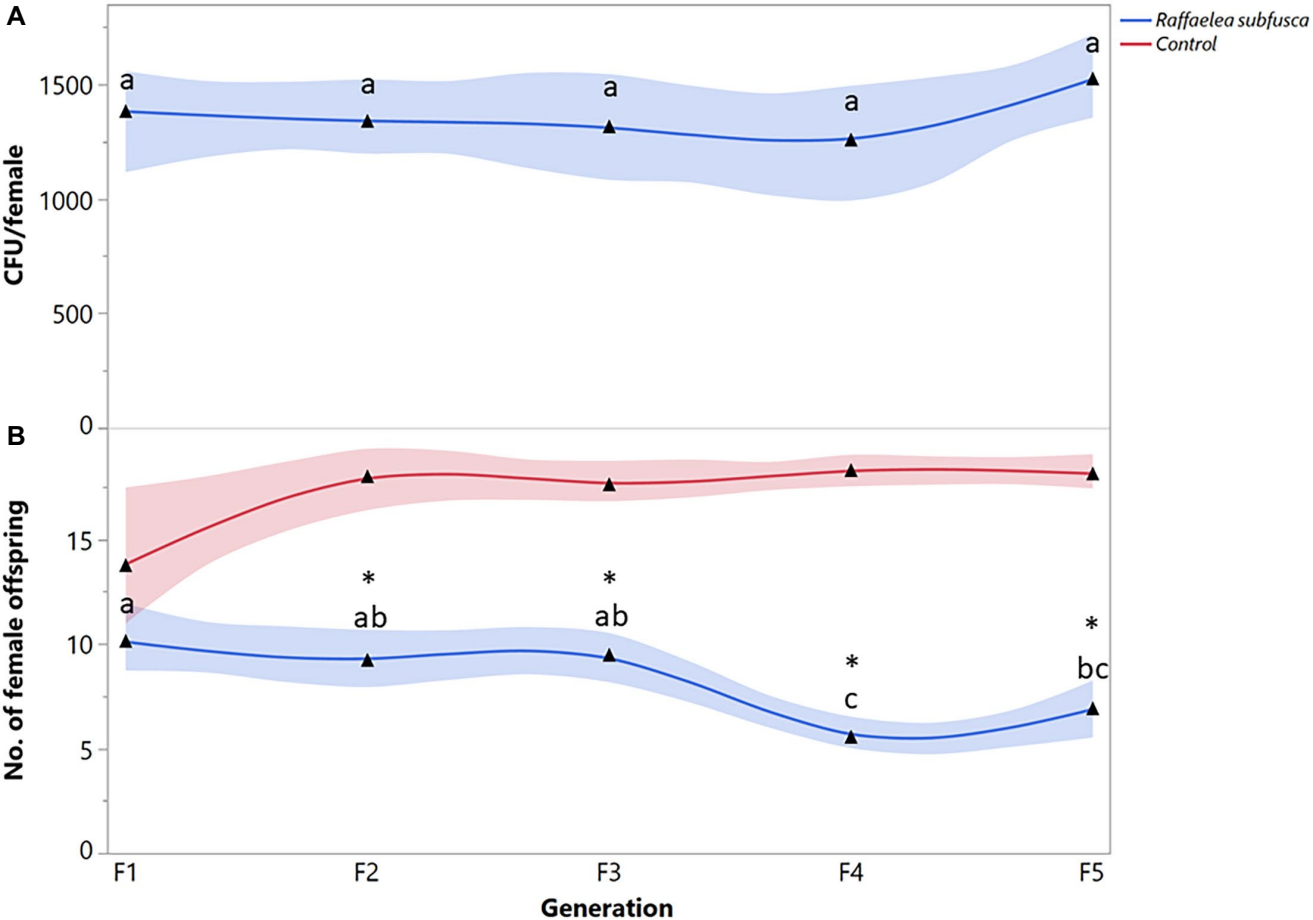
*Xyleborus bispinatus* performance with a single mutualistic fungus (*Raffaelea subfusca*). **(A)** Mean number of CFUs (▲) recovered from single female offspring per generation and **(B)** Mean number of female offspring (▲) produced by single female founder per generation. Same letters within the same color are not significantly different (Tukey HSD, α = 0.05). Asterisks show significant differences in female offspring between fungal symbiont and control within generation (Kruskall-Wallis, α = 0.05).

The number of *R. subfusca* propagules was not significantly different among generations (*F* = 0.8447; df = 4, 63; *p* = 0.6969) (Figure 2A). Females fed on *R. subfusca* diet produced a similar number of female offspring in the first three generations, but reproduction decreased in the last two (*F* = 6.2366; df = 4, 63; *p* = 0.0003) (Figure 2B). Females exposed to a *R. subfusca* diet produced an F_1_ with a similar number of females to the unaltered control (*X^2^* = 2.5452, *p* = 0.1106). However, reproduction in subsequent generations was less than in the control (F_2_: *X^2^* = 18.7822, *p* < 0.0001; F_3_: *X^2^* = 18.9397, *p* < 0.0001; F_4_: *X^2^* = 21.5985, *p* < 0.0001; F_5_: *X^2^* = 25.1267, *p* < 0.0001) (Figure 2B).

**Figure 3:**
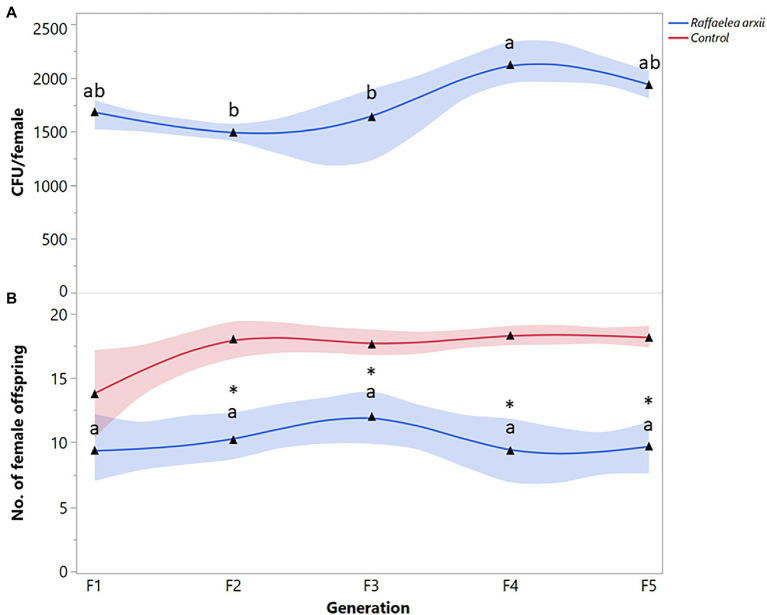
*Xyleborus bispinatus* performance with a single mutualistic fungus (*Raffaelea arxii*). **(A)** Mean number of CFUs (▲) recovered from single female offspring per generation and **(B)** Mean number of female offspring (▲) produced by single female founder per generation. Same letters within the same color are not significantly different (Tukey HSD, α = 0.05). Asterisks show significant differences in female offspring between fungal symbiont and control within generation (Kruskall-Wallis, α = 0.05).

The number of *R. arxii* propagules recovered from *X. bispinatus* varied significantly among generations (*F* = 5.2992; df = 4, 65; *p* = 0.0254). *Raffaelea arxii* CFUs were equally constant in the first three generations but increased in the last two (Figure 3A). *Xyleborus bispinatus* females transitioned to a *R. arxii* diet produced similar number of female offspring during five generations (*F* = 0.7125; df = 4, 65; *p* = 0.5864) (Figure 3B). The F_1_ female offspring of *R. arxii* fed females was similar to the control with unaltered symbiosis (*X^2^* = 2.6894, *p* = 0.1010), but reproduction in subsequent generations was significantly less than in the control (F_2_: *X^2^* = 12.2685, *p* = 0.0005; F_3_: *X^2^* = 11.5424, *p* = 0.0007; F_4_: *X^2^* = 19.3982, *p* < 0.0001; F_5_: *X^2^* = 24.8068, *p* < 0.0001) (Figure 3B).

**Figure 4:**
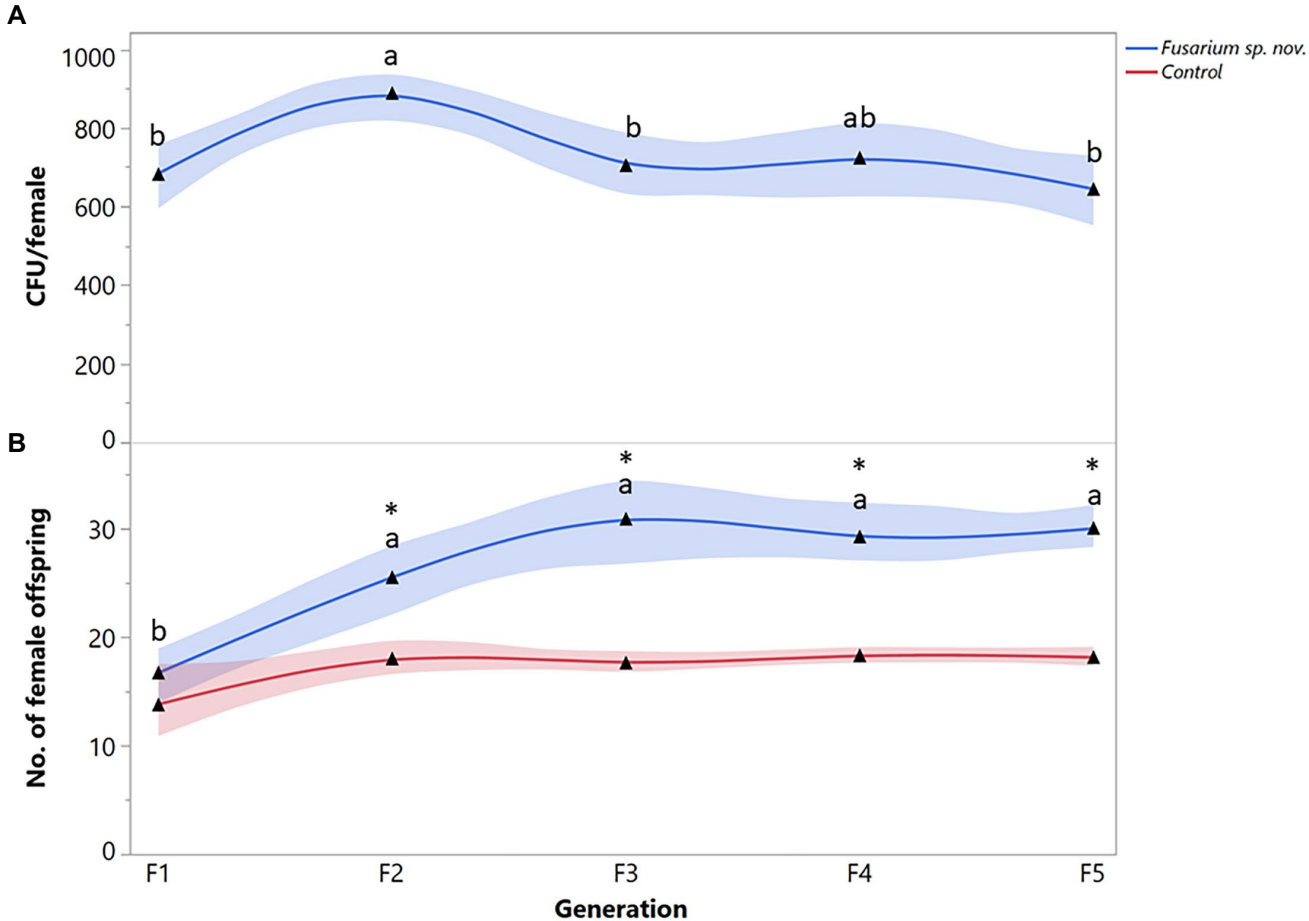
*Xyleborus bispinatus* performance with a single mutualistic fungus (*Fusarium* sp. nov.). **(A)** Mean number of CFUs (▲) recovered from single female offspring per generation and **(B)** Mean number of female offspring (▲) produced by single female founder per generation. Same letters within the same color are not significantly different (Tukey HSD, α = 0.05). Asterisks show significant differences in female offspring between fungal symbiont and control within generation (Kruskall-Wallis, α = 0.05).

Finally, the number of *Fusarium* sp. nov. propagules recovered from *X. bispinatus* varied significantly among generations (*F* = 4.2888; df = 4, 95; *p* = 0.0261). CFUs of *Fusarium* sp. nov. increased from the first to the second generation but decreased in the third generation remaining similar throughout the subsequent generations (Figure 4A). *Xyleborus bispinatus* females that fed on *Fusarium* sp. nov. diet produced a higher number of female offspring in the last four generations than in the first one (*F* = 13.5657; df = 4, 95; *p* < 0.0001) (Figure 4B). In the first generation, *Fusarium* sp. nov. fed females produced similar number of female offspring to the control, but reproduction increased in subsequent generations, significantly more than in the control (Figure 4B).

**Figure 5:**
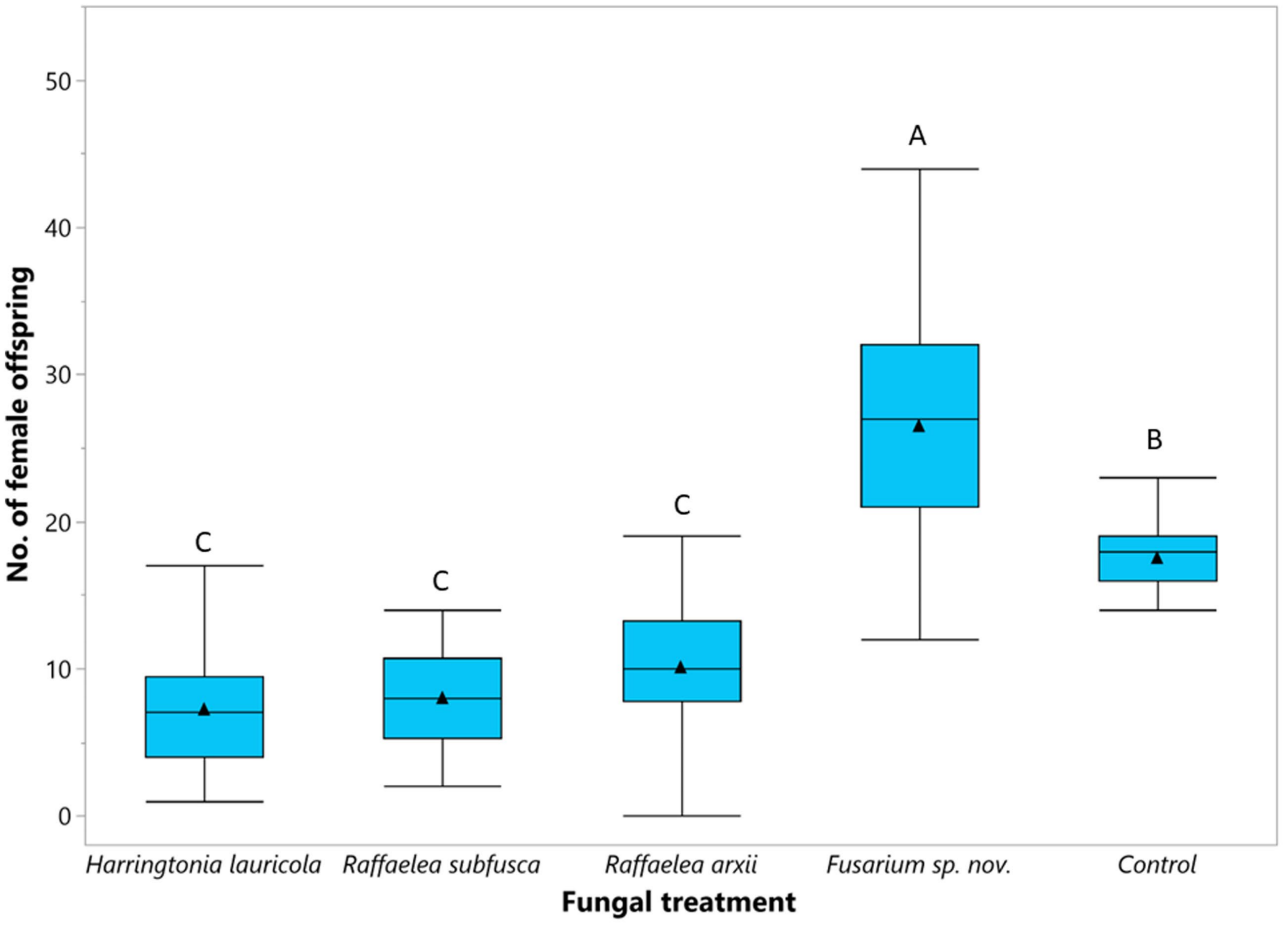
Comparison of the mean number of female offspring (▲) produced by single female founder adapted to single fungal symbiont. Box plots topped with the same letter are not significantly different (Tukey HSD, α = 0.05).

The number of female offspring (*F* = 4.78; df = 4, 311; *p* < 0.0001) varied among fungal treatments (Figure 5). Females fed on a *Fusarium* sp. nov. diet produced the highest number of female offspring (Figure 5). Non-treated beetles (i.e., control) produced fewer individuals than those fed on *Fusarium* sp. nov., but significantly more than beetles reared on *H. lauricola* and the two *Raffaelea* species. There were no significant differences in the number of female offspring among *H. lauricola* and the *Raffaelea* treatments (Figure 5). The number of males ranged between 1 or 2 males per colony and did not vary among treatments.

## 4. Discussion

Laboratory reared *X. bispinatus* exhibits flexibility in its symbiotic associations and can establish stable populations feeding on different fungal diets, including known fungal associates and phylogenetically distant fungi, which are primary symbionts of other ambrosia beetle genera. These changes in fungal diets resulted in persistent mutualistic associations, and some symbionts even increased the beetle’s reproduction. However, ambrosia beetles exhibit high fidelity in their symbiotic relationships with specific groups of fungi under natural conditions. The mechanisms that drive this fidelity have been a matter of active and recent research. In some bark and ambrosia beetles, symbiont fidelity is thought to be mediated by the mycangium (Bracewell and Six, 2015; Mayers et al., 2015, 2020a,b, 2022; Li et al., 2017; Skelton et al., 2019). For instance, *Ambrosiella* fungi thrive in the mycangium of *Xylosandrus* beetles, whereas distantly unrelated fungi are suppressed. The mycangium of *X. bispinatus* does not appear to provide this type of selectivity, allowing mutualistic interactions with distant unrelated fungi. *Xyleborus* spp. have small pre-oral mycangia (Li et al., 2018; Spahr et al., 2020; Mayers et al., 2020a), which are presumed to be less selective or more permissive than larger, more complex mycangia (Mayers et al., 2015, 2020a; Hulcr and Stelinski, 2017; Joseph and Keyhani, 2021). We infer that species with less mycangial selectivity such as *X. bispinatus* are more likely to establish mutualisms with new ambrosia fungi. Interactions with other organisms inhabiting beetle galleries can also play an important role in the fidelity of ambrosia symbiosis. Grubbs et al. (2020) and Nuotclà et al. (2021) suggested that bacteria may selectively exclude non-mutualistic fungi from the galleries and mycangia. In addition, the mycangia may provide an environment and specific nutrients that favor the growth of particular nutritional fungi (e.g., biological screening). The host tree physiology may also affect symbiotic fungi and, thus, their association with a given beetle. Some ambrosia beetles target stressed trees producing ethanol while others do not. Ethanol-enriched wood favors the growth of particular nutritional symbionts while suppressing others. This suggests that ethanol content in wood may influence the symbiont composition and thus the likelihood of novel symbiont acquisition (Ranger et al., 2018; Cavaletto et al., 2021, 2022; Lehenberger et al., 2021b). However, such selective effects of substrate were not present in our artificial medium and may influence the effects of particular fungi on beetle fitness *in vivo*.

Some ambrosia beetles can use one or more closely related fungi (Skelton et al., 2019; Carrillo et al., 2020a) as nutritional food sources. *Raffaelea* species are common partners of *Xyleborus* species (Harrington et al., 2010; Campbell et al., 2016; Cruz et al., 2018, 2019; Saucedo-Carabez et al., 2018); therefore, the survival and reproduction of *X. bispinatus* on *R. subfusca* and *R. arxii* diets was expected. By contrast, *X. bispinatus* reared on *H. lauricola* declined progressively and stopped reproducing after five generations. This result suggests that *H. lauricola* was a suboptimal food source for *X. bispinatus*, which contrasts with previous reports indicating that this fungus enables high fecundity rates and significant brood production in this beetle (Saucedo et al., 2017). Our results also suggest that *X. bispinatus* benefits from feeding upon multiple ambrosia fungi. Colonies fed singly on *H. lauricola* or *Raffaelea* spp. had fewer female offspring than colonies from the control treatment (Figures 1B, 2B, 3B). In agreement with these results, a previous study demonstrated that wild *X. bispinatus* increased reproduction when *H. lauricola* was added to their diet (Menocal et al., 2018a). Altogether, the available information suggests that *X. bispinatus* benefits from the new partnership with *H. lauricola*, but it is unlikely to use it as its primary symbiont.

Mutualisms between *Euwallacea* beetles and *Fusarium* spp. are well recognized (Freeman et al., 2012, 2016; Carrillo et al., 2016, 2020b; O’Donnell et al., 2016; Kendra et al., 2022), but associations between *Fusarium* spp. and other ambrosia beetles are presumed to be incidental and non-mutualistic (Biedermann et al., 2013; Kostovcik et al., 2015; Bateman et al., 2016; Biedermann, 2020). In our study, *X. bispinatus* engaged in a persistent mutualism with *Fusarium* sp. nov. [currently >20 generations – data not shown]. This fungus sustained significantly more female offspring than any other single fungus or the unmanipulated colonies with several fungal symbionts (Figure 5). This result is noteworthy because *Fusarium* spp. belongs to a distant unrelated taxonomic order (Hypocreales) than most other ambrosia fungi (Ophiostomatales). Interestingly, *Fusarium solani* (Norris and Baker, 1967, 1968) and *Fusarium* sp. (AF-9) (Kasson et al., 2013) were reported as mycangial fungal partners of *Xyleborus ferrugineus* Fabricius. Moreover, *X. ferrugineus* and *X. bispinatus* were considered synonyms until Atkinson et al. (2013) recognized them as separate species. These results and our findings suggest that both beetles may exhibit mutualisms with *Fusarium* spp. and that these symbiotic relationships could be more common than previously presumed.

Our results suggest that exotic ambrosia beetles can induce changes in resident ambrosia beetle communities and their symbionts. In their native range, ambrosia beetles are rooted in a network of interactions with native symbiotic fungi, including endophytes, plant pathogens, and mutualists (Simmons et al., 2016; Carrillo et al., 2019). In invaded areas, ambrosia beetles face novel environments, new host opportunities, and potentially novel symbionts (Rassati et al., 2019). In some cases, invasive beetles may not survive *in-or* adapt *to-a* new environment (e.g., *X. glabratus* in avocado) but their fungi (e.g., *H. lauricola*) can establish novel partnerships with resident ambrosia beetles (Carrillo et al., 2014; Ploetz et al., 2017; Cruz et al., 2021). The symbionts’ ecology could play a major role in developing novel mutualisms. Most ambrosia beetle symbionts only grow in the xylem-sapwood in close proximity to the beetle galleries and inside the beetle mycangia. Unlike most *Raffaelea* species, *H. lauricola* displays systemic growth in its lauraceous hosts (Lynch et al., 2012; Ploetz et al., 2012; Campbell et al., 2017). This could be a significant contributing factor for the lateral transfer of this fungus to multiple ambrosia beetles in areas of recent invasion in the USA. For ambrosia fungi with localized growth, sympatric beetle breeding and natal gallery overlapping may be required for fungal exchange. Under these circumstances, competitive fungi displaying fast growth and the ability to nourish ambrosia beetles can outcompete other symbionts and engage in new partnerships. In our experiment, *Fusarium* sp. nov. appeared to colonize the rearing substrate faster than other fungi. *Fusarium* sp. nov. may be more nutritious or better at translocating elements from the rearing plug to its fruiting bodies. Fungal mutualists translocate essential elements such as calcium (Ca), nitrogen (N), phosphorous (P), potassium (K), magnesium (Mg), manganese (Mn), and sulfur (S), through their hyphae from the sapwood to fruiting structures in the galleries (Six and Elser, 2019; Lehenberger et al., 2021a). These elements are essential for fungal and beetle growth (Filipiak et al., 2016). A better understanding of the nutritional quality and growth patterns of ambrosia fungi may help identify species with invasive potential and predict novel ambrosia symbioses.

In conclusion, species such as *X. bispinatus* with fungus-feeding plasticity are likely to establish new symbiotic relationships with exotic ambrosia fungi like *H. lauricola*. However, our study was conducted under simplified laboratory conditions. Interactions with multiple abiotic (e.g., temperature, relative humidity) and biotic (plant-secondary compounds and other microbial players) factors under natural conditions may lead to other fitness effects and more complex associations. The ecology of the fungal symbionts may also play an important role in novel ambrosia symbiosis. Further experiments on ambrosia beetle symbiosis may help identify patterns and characteristics that make some species more likely to become invasive.

## Data availability statement

The original contributions presented in the study are included in the article, further inquiries can be directed to the corresponding authors. The data (i.e., fungal sequences) presented in the study were uploaded to the NCBI GenBank (Home - Nucleotide - NCBI (https://www.ncbi.nlm.nih.gov) database and are publicly available (Accession numbers are included in Table 1).

## Author contributions

OM, LC, and DC conceived and designed the study. OM and LC performed the experiments and collected the data. OM analyzed the data. PK and MB contributed with reagents/materials/analysis tools. OM wrote the initial draft of the manuscript. LC and DC provided feedback and comments on previous versions of the manuscript. PK and DC reviewed and edited the final version of the manuscript. All authors contributed to the article and approved the submitted version.

## Funding

This research was funded by NIFA grant 2015–51181-24257 to Daniel Carrillo and by a Non-Assistance Cooperative Agreement between the USDA-ARS and the University of Florida (number: 58-6038–8-004).

## Conflict of interest

The authors declare that the research was conducted in the absence of any commercial or financial relationships that could be construed as a potential conflict of interest.

## Publisher’s note

All claims expressed in this article are solely those of the authors and do not necessarily represent those of their affiliated organizations, or those of the publisher, the editors and the reviewers. Any product that may be evaluated in this article, or claim that may be made by its manufacturer, is not guaranteed or endorsed by the publisher.
